# A Multidisciplinary Rehabilitation Approach for a Patient With Diffuse Large B-cell Lymphoma and Bone Metastasis: A Case Report

**DOI:** 10.7759/cureus.60713

**Published:** 2024-05-20

**Authors:** Gaku Watanabe, Yukiyo Shimizu, Yuki Mataki, Kayo Tokeji, Yasushi Hada

**Affiliations:** 1 Rehabilitation Medicine, University of Tsukuba Hospital, Tsukuba, JPN; 2 Rehabilitation Medicine, Faculty of Medicine, University of Tsukuba, Tsukuba, JPN

**Keywords:** rehabilitation therapy, lymphoma, multidisciplinary approach, pathological fracture, skeletal-related events, bone metastasis

## Abstract

Cancer is often accompanied by bone metastasis, which may lead to skeletal-related events (SREs), such as pain, hypercalcemia, pathological fractures, spinal cord compression, orthopedic surgical intervention, and palliative radiation directed at the bone. Herein, we report the case of a 75-year-old female patient diagnosed with diffuse large B-cell lymphoma (DLBCL) with bone metastasis and a pathological fracture of the right iliac bone. The management strategy and follow-up were determined by a multidisciplinary cancer board comprising physicians, physiatrists, orthopedic surgeons, radiologists, and rehabilitation therapists. A conservative approach was chosen, incorporating a bone-modifying agent and weight-bearing restrictions for the right leg, along with rehabilitation therapy and post-discharge support. A multidisciplinary rehabilitation approach for two months enabled the patient to walk independently upon discharge. She maintains her activities of daily living (ADL) for over six months after discharge without any skeletal issues. This case highlights the effectiveness of a multidisciplinary approach in managing bone metastasis or involvement in patients with lymphoma.

## Introduction

Advancements in cancer treatment modalities, including surgery, radiation therapy, chemotherapy, hormone therapy, and molecular-targeted therapy, have demonstrably improved patient survival rates [1-3]. However, the prevalence of bone metastasis as a comorbidity has consequently risen [4]. Bone metastases can lead to a spectrum of skeletal-related events (SREs), such as pain, hypercalcemia, pathological fractures, spinal cord compression, orthopedic surgical intervention, and palliative radiation directed at the bone. These SREs often significantly impair patients’ activities of daily living (ADLs), thereby negatively impacting both cancer treatment adherence and quality of life (QoL) [5,6]. Rehabilitation therapy is reported to be effective for patients with bone metastasis, or SREs [7,8]. Furthermore, effective management of bone metastases is crucial, and previous studies have reported the usefulness of a multidisciplinary approach in this regard [6,9]. Lymphoma is the most common hematological malignancy worldwide. The five-year relative survival rate is approximately 68%, with a mortality rate of 11.2 deaths per 100,000 people in Japan [10]. It is common among older individuals, with the majority of cases being non-Hodgkin lymphoma (NHL). Diffuse large B-cell lymphoma (DLBCL) is the most frequent subtype of NHL and accounts for approximately 30% of NHL cases in Japan [11]. It includes morphologically and molecularly heterogeneous disease subtypes [12]. Patients with lymphoma often experience bone metastasis or bone involvement, as well as other cancers [13]. Rehabilitation in patients with lymphoma is reported to have beneficial effects on physical performance and QoL [14]. However, evidence of rehabilitation and a multidisciplinary approach in patients with bone metastasis or bone involvement in lymphoma is weak. This case report presents the application of a multidisciplinary rehabilitation approach to treating a patient with DLBCL and associated bone metastasis. Verbal informed consent was obtained from the patient for the publication of this case report.

## Case presentation

**Figure 1:**
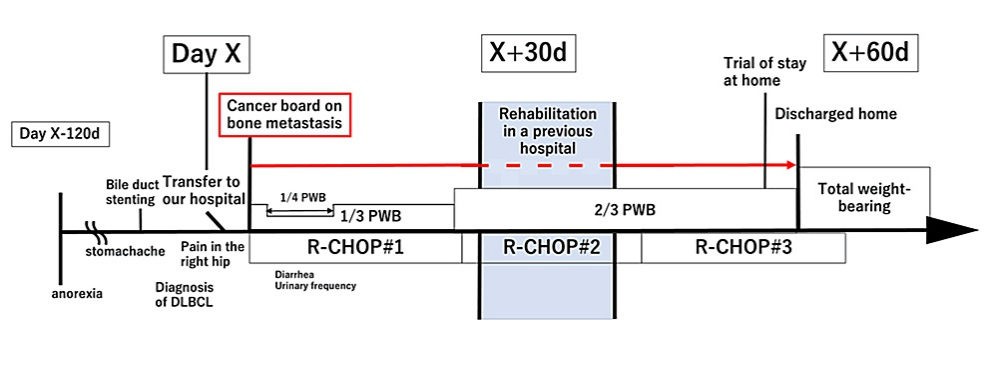
Clinical course. The figure illustrates the clinical course of the patient from the onset of symptoms. She was transferred to the previous hospital to continue her rehabilitation in the blue segment period.

**Figure 2:**
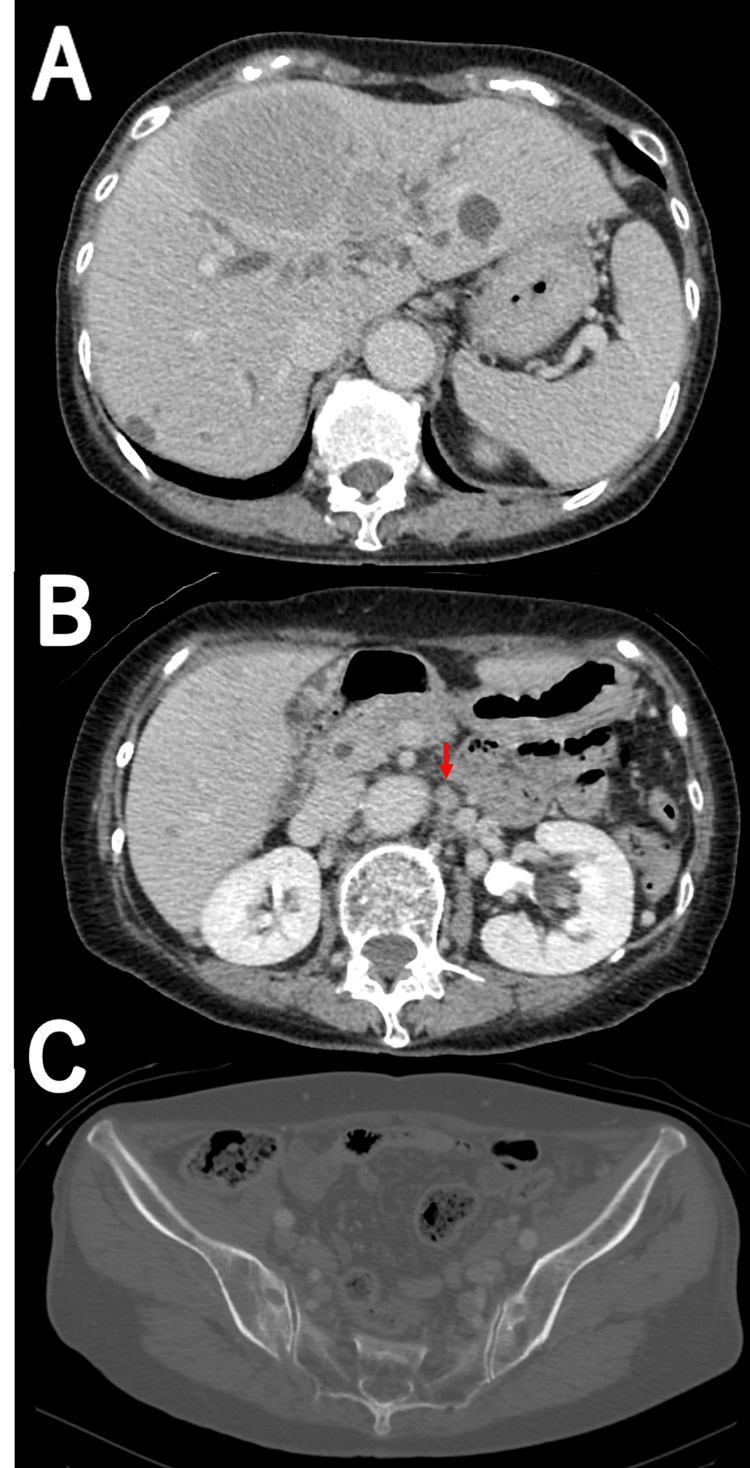
CT scan from a previous hospital. (A) CT scan shows multiple masses in the liver and dilated intrahepatic bile ducts; (B) para-aortic lymph nodes are enlarged (red arrow); (C) the cortex of the right iliac bone appears continuous.

A 75-year-old female presented to a local hospital with a four-month history of anorexia and a recent stomach ache (Figure 1). She was fully independent in all ADLs, including instrumental tasks, and lived alone in a single-story house before hospitalization. Her medical history was significant for osteoporosis, postoperative uterine fibroids, hepatitis C, and constipation. Her medications included alendronic acid and magnesium oxide. She had no family history of malignancies. Computed tomography (CT) revealed multiple liver masses and enlarged mesenteric and retroperitoneal lymph nodes (Figure 2). The patient underwent bile duct stenting and a biopsy and was diagnosed with primary hepatic lymphoma based on the pathology of DLBCL. Nine days after diagnosis, the patient was transferred to our hospital for multimodal treatment for DLBCL.

On the day of transfer (day X), we received a rehabilitation referral and conducted an initial assessment. Upon admission, the patient was alert and complained of pain in the right hip with an intensity of 8/10 on the numeric rating scale (NRS). The pain was continuous, exacerbated with movement, and radiated to the right lower extremity. She exhibited tenderness in the sciatic region and weakness (manual muscle testing [MMT] grade 3-4/5) in the proximal muscles of the right lower extremity affected by pain. The straight leg raise test on the right leg was limited to 45°. The range of motion showed no restriction. Her ambulation was limited to short distances with a T-cane, and she relied heavily on the cane while experiencing significant pain in her lower limbs.

**Figure 3:**
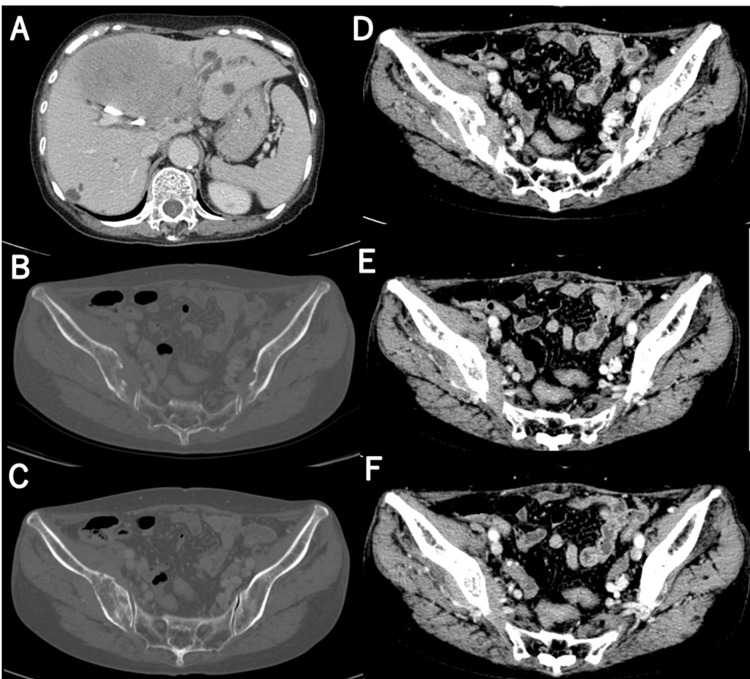
CT scan on admission. (A) CT scan shows the multiple mass in the liver and a biliary stent; (B) it also revealed the osteolytic change; (C) a pathological fracture of the right iliac bone near the sacroiliac joint; and (D-F) a mass of soft tissue density around it and extended continuously to the right ischial foramen.

Her height and body weight were 159 cm and 48.4 kg, respectively. Comprehensive blood tests were performed, including a complete blood count and biochemical analysis. Our findings indicated elevated levels of lactate dehydrogenase (LDH) (1,022 U/L), alkaline phosphatase (ALP) (1,726 U/L), gamma-glutamyl transferase (γGTP) (510 U/L), c-reactive protein (CRP) (2.99 mg/dL), and soluble interleukin-2 receptor (sIL2-receptor) (2,814 U/mL), along with decreased albumin levels (3.4 g/dL). Serum calcium levels remained within the normal range (9.1 mg/dL). The detailed blood test results are presented in Table 1. A whole-body CT (Figure 3) revealed osteolytic changes in the right iliac bone near the sacroiliac joint with a pathological fracture. A surrounding soft tissue mass extended towards the right ischial foramen, likely contributing to the patient’s pain. Based on these findings, she was diagnosed with Ann Arbor stage IVB DLBCL and assigned a National Comprehensive Cancer Network International Prognostic Index (NCCN-IPI) of eight.

**Table 1:** Results of the comprehensive blood test. AST: aspartate aminotransferase; ALT: alanine aminotransferase; γGTP: gamma-glutamyl transferase; LDH: lactate dehydrogenase; ALP: alkaline phosphatase; BUN: blood urea nitrogen; CRP: C-reactive protein; IP: inorganic phosphate.

Investigations	Normal	Patient's report	Comments
Hemoglobin	12–16 g/dL	13.5	Normal
Hematocrit	33.5–45%	41.8	Normal
Total leukocyte count	4–9 × 10^³^μL	7.3	Normal
Neutrophils	45–55%	78	Increase
Lymphocytes	25–45%	11	Decrease
Platelets	150–350 × 10^³ ^μL	346	Normal
BUN	8–20 mg/dL	14.5	Normal
Creatinine	0.47–0.79 mg/dL	0.43	Decrease
Total bilirubin	0.3–1.2 mg/dL	1.5	Increase
Direct bilirubin	0–0.4 mg/dL	0.5	Increase
AST	8–38 U/L	62	Increase
ALT	4–44 U/L	34	Normal
γGTP	8–38 mg/dL	510	Increase
LDH	106–322 U/L	1,022	Increase
ALP	104–338 U/L	1,726	Increase
Albumin	3.8–5.3 g/dL	3.4	Decrease
CRP	0–0.2 mg/ dL	2.99	Increase
Calcium	8.6–10.1 mg/dL	9.1	Normal
IP	2.7–4.5 mg/dL	4.3	Normal
Soluble IL-2 receptor	157–474 U/mL	2,814	Increase

**Figure 4:**
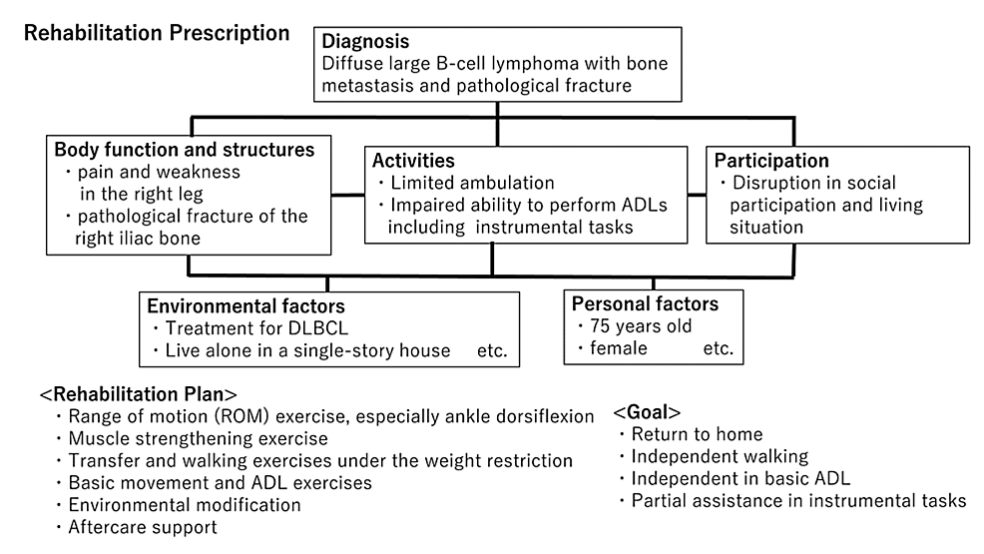
Rehabilitation prescription based on the International Classification of Functioning, Disability and Health (ICF).

Based on the diagnosis of DLBCL with bone metastasis and pathological fracture, we prescribed physical therapy (Figure 4). Initially, we directed the physiotherapist to restrict the rehabilitation program with a primary focus on shoulder and ankle range-of-motion exercises, as well as floor activity training, owing to SREs. On day X+1, R-CHOP chemotherapy (comprising rituximab, cyclophosphamide, doxorubicin, vincristine, and prednisolone) was initiated. Additionally, a multidisciplinary cancer board meeting involving physicians, physiatrists, orthopedic surgeons, radiation oncologists, and rehabilitation therapists was held to discuss the patient’s condition and management of bone metastasis, including surgical, radiation, and rehabilitation options. A conservative approach was adopted in the present case, with continued administration of the bone-modifying agent alendronate. Furthermore, one-third of partial weight-bearing on the right leg was permitted, contingent upon whether pain was controlled.

As a result of the discussion on the cancer board, it was recommended to the patient that she use a wheelchair. She simultaneously started transfer training, from bed to wheelchair, from wheelchair to toilet, and vice versa. Moreover, she practiced weight distribution using a weight scale to limit excessive weight on her right leg and started gait training with a physical therapist. The side effects of chemotherapy, such as diarrhea and frequent urination, led to increased transfers between the bed and wheelchair, which exacerbated her pain. Although weight-bearing on the right leg was temporarily reduced to one-quarter in accordance with the pain augmentation, her condition soon improved. A CT scan conducted three weeks after treatment initiation showed a reduced liver mass size, bony callus formation at the fracture site, and pain reduction to 1/10 on the NRS. Consequently, weight-bearing on the right leg was increased to two-thirds, and gait training progressed to using a pickup walker. Initially, she feared walking but gradually gained confidence through training.

**Figure 5:**
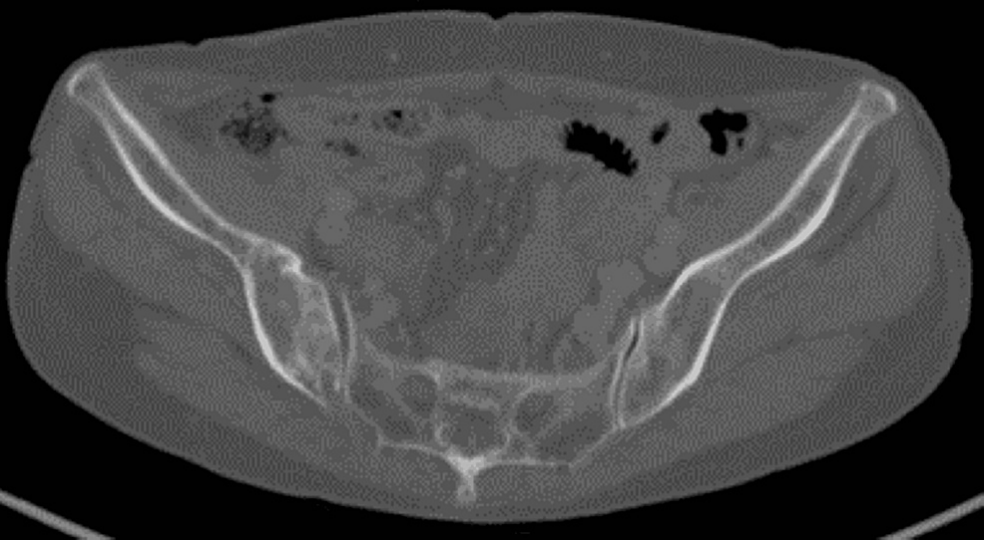
CT scan taken at six months after discharge. Osteosclerosis and fusion of the right iliac bone near the sacroiliac joint are visible.

Following intravenous administration of the second cycle of R-CHOP chemotherapy on day X+23, the patient underwent a three-week rehabilitation program at a previous hospital owing to limitations in returning home directly. On day X+44, she was transferred back to our hospital for the third cycle of R-CHOP chemotherapy. She began gait training using a T-cane, and preparations for discharge commenced. To prepare for home discharge, our multidisciplinary rehabilitation team discussed the implementation of long-term care insurance and home modifications. With the assistance of a medical social worker, she applied for care insurance and proceeded to obtain a shower chair and modify her home environment to eliminate steps. After completing the third cycle of R-CHOP chemotherapy, a successful homestay trial confirmed her ability to manage indoor ADLs. She arranged for a substitute service to handle her shopping. It was planned that she would receive support for instrumental ADLs, such as housework and outings, from her daughter-in-law, who lived near her house. On day X+56, she was discharged, ambulating home independently with a T-cane. The patient underwent monthly follow-ups for bone metastases following discharge. She has maintained her ADLs for over six months without any skeletal issues (Figure 5).

## Discussion

The most important finding of this report was the potential benefits of a multidisciplinary rehabilitation approach for patients with lymphoma associated with bone metastasis or bone involvement.

Patients with cancer often experience a decline in physical function owing to disuse caused by medication side effects, fatigue, and other factors. Rehabilitation interventions can help patients maintain and improve physical function and ADLs and alleviate the side effects related to cancer therapy [15-17]. Rehabilitation for patients with blood malignancies, including lymphoma, also improves their physical function, ADLs, and QoL [14]. Cancer rehabilitation empowers individuals to live with dignity throughout all phases of the disease, from diagnosis to end-of-life care. Risk management, including that of bone metastasis, is an essential component of rehabilitation.

Advanced stages of cancer are often accompanied by bone metastasis. Breast, prostate, lung, and thyroid cancers, as well as multiple myeloma, are especially associated with bone metastases [9,18,19]. Bone metastasis can result in morbidities such as cancer-induced bone pain, hypercalcemia, pathological fractures, and spinal cord compression, collectively known as SREs. These SREs can further aggravate patients’ QoL and impair their ability to perform ADLs independently [18,19]. Treatment options for bone metastasis include radiation therapy, surgery, and the use of bone-modifying agents. Studies have suggested the effectiveness of a multidisciplinary approach in managing bone metastasis [6,9].

Lymphoma is the most common hematological malignancy and is prevalent among older individuals. The five-year relative survival rate is approximately 68%, with a mortality rate of 11.2 deaths per 100,000 people in Japan [10]. The majority of cases are NHL, with DLBCL being the most frequent subtype, accounting for approximately 30% of NHL cases in Japan [14]. Approximately 7-10% of patients with lymphoma have skeletal involvement [13], primarily secondary to systemic lymphoma disease [1,20]. The common sites of bone involvement are the spine and pelvis [4,20]. In patients with vertebral involvement, there is a risk of paralysis due to spinal cord compression, whereas pelvic involvement may require weight restrictions, and rehabilitation involving motion instruction becomes necessary. Multidisciplinary approaches similar to those for bone metastasis of solid tumors and multiple myeloma are also considered effective in the management of bone metastases secondary to lymphoma. However, to the best of our knowledge, reports on a multidisciplinary approach for managing bone involvement in patients with lymphoma are scarce, and its efficacy remains unclear.

Here, we report a multidisciplinary approach for managing a patient with DLBCL, bone metastasis, and a pathological fracture. A conference with physicians, physiatrists, orthopedic surgeons, radiologists, and rehabilitation therapists was held to discuss appropriate management strategies for bone metastasis. During the conference, we shared information regarding the patient’s physical condition, prognosis, and domestic environment, enabling us to promptly determine management, including treatment options such as surgery and radiation therapy, rehabilitation strategies, aftercare support, and follow-up plans. Additionally, the patient received rehabilitation therapy under appropriate risk management, preventing further deterioration of her ADLs and QoL. Further, she was discharged with adequate aftercare support, including utilization of a long-term care insurance system, which is mandatory for individuals aged 40 and above in Japan. This case underscores the potential benefits of an integrated multidisciplinary rehabilitation approach in patients with lymphoma complicated by bone metastasis or bone involvement. This approach may have contributed to the maintenance of her ADLs and QoL, facilitating the patient’s discharge with supportive aftercare. However, given the inherent limitations of case reports and the need for a more robust evidence base, our findings should be interpreted with caution. We advocate for further research, including larger-scale studies and long-term follow-up, to validate the observed benefits and refine the management strategies for patients with lymphoma accompanied by bone involvement. Such research is essential to establishing evidence-based guidelines that can be widely applied in clinical practice to improve patient outcomes.

## Conclusions

We reported a case of DLBCL with bone metastasis, highlighting the potential effectiveness of a multidisciplinary rehabilitation approach in managing patients with lymphoma and bone involvement. Through detailed documentation of interventions and outcomes, our aim is to provide insight into the potential benefits of a coordinated, interprofessional approach to care for such patients. However, it is imperative to acknowledge the need for further research and the accumulation of additional cases to substantiate the efficacy of this approach.
